# Five new *Sinopoda* species (Araneae, Sparassidae) from China and Thailand

**DOI:** 10.3897/zookeys.1012.59854

**Published:** 2021-01-26

**Authors:** Ziyi Wang, Wei Liang, Shuqiang Li

**Affiliations:** 1 Ministry of Education Key Laboratory for Ecology of Tropical Islands, Hainan Normal University, 571158, Haikou, China Hainan Normal University Haikou China; 2 Institute of Zoology, Chinese Academy of Sciences, 100101, Beijing, China Institute of Zoology, Chinese Academy of Sciences Beijing China

**Keywords:** Biodiversity, distribution, huntsman spiders, taxonomy

## Abstract

Five new species of the huntsman spider genus *Sinopoda* Jäger, 1999 are described: *S. hongruii* Wang & Li, **sp. nov.** (♂♀, forest in Anhui, China), *S. jiangzhou* Wang & Li, **sp. nov.** (♂♀, cave in Guangxi, China), *S. saiyok* Wang & Li, **sp. nov.** (♀, cave in Kanchanaburi, Thailand), *S. yanjin* Wang & Li, **sp. nov.** (♀, forest in Yunnan, China), and *S. yanzi* Wang & Li, **sp. nov.** (♂♀, cave in Hunan, China). A distribution map of the new species is provided.

## Introduction

Sparassidae Bertkau, 1872 are small to large spiders with laterigrade legs. The genus *Sinopoda* was established by Jäger in 1999 and belongs to the subfamily Heteropodinae Thorell, 1873 (Jäger 1999). The genus can be distinguished from other huntsman spiders by the presence of an embolic apophysis and a membranous conductor in the male palp, and by the special internal ducts in female vulva (Jäger 1999; Liu et al. 2008; Zhang et al. 2015). *Sinopoda* is the fourth largest genus of Sparassidae, with 126 species from Asia reported: 65 from China, 16 from Laos, 12 from Malaysia, 11 from Japan and South Korea, nine from Thailand, five from Myanmar, four from Indonesia, three from Vietnam, and one from India (WSC 2020; Li 2020).

*Sinopoda* are non-web building spiders, living in leaf litter, rock crevices, caves, and on tree bark (Jäger 1999, 2012). *Sinopoda* spiders are difficult to collect in the field due to their cryptic life style and nocturnality; thus, about 40% of the species are known only from a single sex. In this paper, all five new *Sinopoda* species, including two known from females and three from both sexes, are found in typical habitat: three from caves and two from rock crevices in forests.

## Methods

All the specimens were collected, preserved in 75% ethanol, and examined and measured with a Leica M205C stereomicroscope. After dissection of male palps and the epigynes, images were made with an Olympus C7070 wide zoom digital camera (7.1 megapixels) mounted on an Olympus BX51 compound light microscope. Images of the spiders’ bodies were taken with an Olympus C7070 camera mounted on an Olympus SZX12 dissecting microscope. The epigynes were cleaned and treated in trypsin and, if necessary, in a boiling solution of potassium hydroxide (KOH) before being transferred to 75% ethanol for imaging. All images were assembled using Helicon Focus v. 6.7.1 software.

All measurements are in millimeters. Leg formula, spination, and measurements of palps and legs follow Jäger (2012). The point of origin of the embolus and conductor are given as “clock positions” on the left palps in ventral view.

Abbreviations used in the text:

**ALE** anterior lateral eyes;

**AME** anterior median eyes;

**AW** anterior width of prosoma;

**CH** clypeus height;

**dRTA** dorsal branch of RTA;

**E** embolus;

**EA** embolic apophysis;

**EP** epigynal pockets;

**FB** fusion bubble;

**FD** fertilization ducts;

**LF** lateral furrow;

**LL** lateral lobes;

**LS** lobal septum;

**OL** opisthosoma length;

**OW** opisthosoma width;

**PL** prosoma length;

**PW** prosoma width;

**PLE** posterior lateral eyes;

**PME** posterior median eyes;

**PP** posterior part of internal duct system;

**SP** spermophor;

**RTA** retrolateral tibial apophysis;

**vRTA** ventral branch RTA;

**I, II, III, IV** legs I to IV.

All material is deposited in the Institute of Zoology, Chinese Academy of Sciences (**IZCAS**) in Beijing, China.

## Taxonomy

### Family Sparassidae Bertkau, 1872

**Subfamily Heteropodinae Thorell, 1873**

#### Genus *Sinopoda* Jäger, 1999

##### Sinopoda hongruii

Taxon classificationAnimaliaAraneaeSparassidae

Wang & Li sp. nov.

256A4B51-8D0E-5FA9-ABEB-B8F202F8753A

http://zoobank.org/A9AC1661-6533-4AE3-AFC4-D53660887306

**Figure 1.**
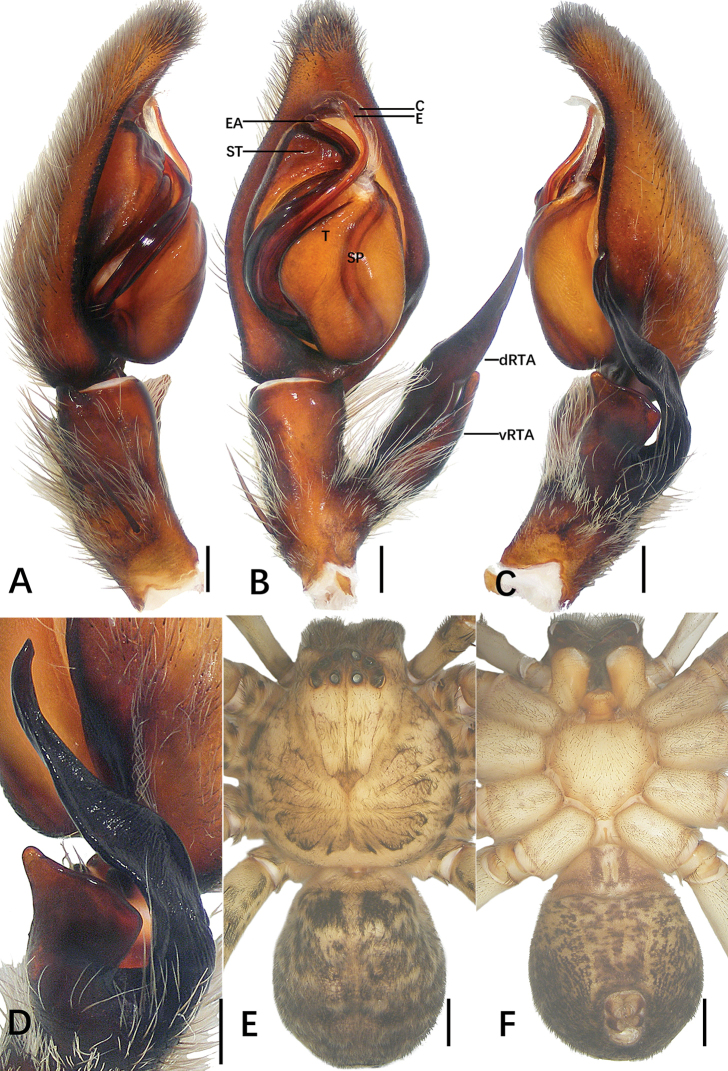
*Sinopoda hongruii* sp. nov., holotype male from Yefu Mountain National Forest Park **A–C** left palp (**A** prolateral **B** ventral **C** retrolateral) **D** retrolateral view of RTA**E, F** habitus (**E** dorsal **F** ventral). Abbreviations: C conductor, dRTA dorsal branch of retrolateral tibial apophysis, E embolus, EA embolic apophysis, SP spermophor, ST subtegulum, T tegulum, vRTA ventral branch of retrolateral tibial apophysis. Scale bars: 0.5 mm (**A–D**); 2 mm (**E, F**).

**Figure 2.**
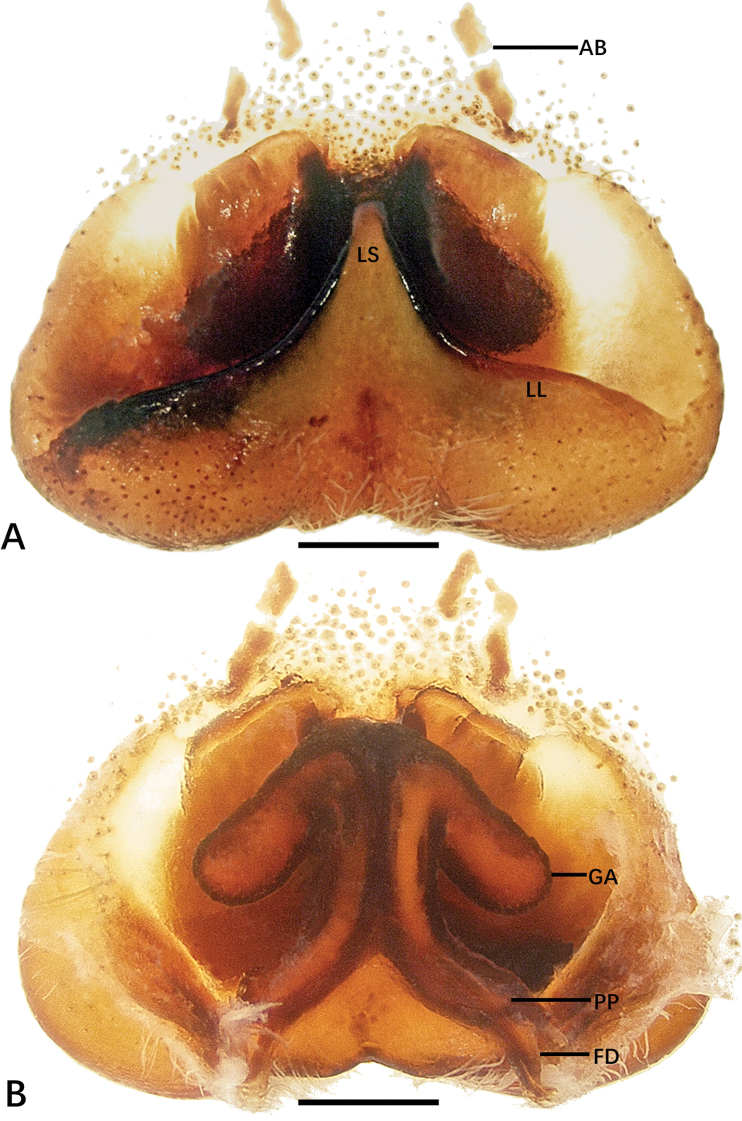
*Sinopoda hongruii* sp. nov., holotype female from Yefu Mountain National Forest Park **A** epigyne **B** vulval. Abbreviations: AB anterior bands, FD fertilization ducts, GA glandular appendages, LL lateral lobes, LS lobal septum, PP posterior part of internal duct system. Scale bars: 0.5 mm.

Figs 1A–F , 2A, B , 9A, B , 10

###### Material examined.

***Holotype*** ♂ (IZCAS-Ar41604), China, Anhui Province, Lujiang County, Yefu Mountain National Forest Park; 31.5674°N, 117.5593°E; 170 m; 3 Jul. 2018; Hongrui Zhao leg. ***Paratypes*** 2 ♀ (IZCAS-Ar41605, IZCAS-Ar41606); China, Anhui Province, Lujiang County, Yefu Mountain National Forest Park; 31.2694°N, 117.2703°E; 50 m; 5 Sept. 2020; Ziyi Wang leg.

###### Diagnosis.

The male of this new species resembles the male of *Sinopoda tengchongensis* Fu & Zhu, 2008 (Fu and Zhu 2008: 63, figs 1–5; Grall and Jäger 2020: 66, fig. 43a–c) in having the analogous conductor and embolus, but the new species can be recognized by the following: the distal part of vRTA is wider than the basal part in retrolateral view in this new species (Fig. 1A–D) but equal in width in *S. tengchongensis*; the tip of the embolus apophysis is flagelliform in the new species but flat in *S. tengchongensis*. The females of this new species are similar to *Sinopoda aequalis* Zhong, Jäger, Chen & Liu, 2019 (Zhong et al 2019: 8, figs 4D, E, 6A–D) in having the anterior part of the internal ducts similar and *S. tengchongensis* Fu & Zhu, 2008 (Fu and Zhu 2008: 63, figs 1–5; Grall and Jäger 2020: 66, fig. 43a–c) in having similar lateral lobes, but can be recognized by the following: the lobal septum is sharper than in *S. aequalis* and *S. tengchongensis*; the new species has blunt, swollen glandular appendages but in *S. aequalis* the glandular appendages are slender and longer; the posterior part of internal duct system as wide as the middle part of internal ducts (Fig. 2A, B) in the new species, while the posterior part of internal duct system swollen and much wider than the internal ducts in *S. aequalis*; the internal duct system is fused along whole median line in the new species but the anterior part is not fused in *S. tengchongensis*.

###### Description.

**Male** (***holotype***, IZCAS-Ar41604) **Measurements**: PL 9.3, PW 8.8; AW 3.8; OL 9.9, OW 5.5. ***Eyes***: AME 0.40, ALE 0.42, PME 0.39, PLE 0.59, AME-AME 0.37, AME-ALE 0.09, PME-PME 0.39, PME-PLE 0.29, AME-PME 0.47, ALE-PLE 0.26, CHAME 0.27, CHALE 0.25. ***Palp***: 12.34 (4.22, 2.04, 2.17, –, 3.91). ***Legs***: I 34.06 (10.24, 3.55, 9.98, 6.84, 3.45); II 38.95 (10.81, 3.58, 10.62, 10.49, 3.45); III 28.77 (8.57, 3.45, 7.61, 6.65, 2.49); IV 26.99 (7.29, 2.68, 7.55, 6.59, 2.88). Leg formula: II-I-III-IV. **Spination: *Palp***: 131 101 – 1100. ***Legs***: Fe I–IV 232 Pa I–IV 101, Ti I–III 2326, IV 2337, Mt I–III 0004, IV 2025. ***Chelicerae***: Furrow with four anterior teeth, four posterior teeth, and 27 denticles.

***Palp***: as in diagnosis. The ratio of the length of the cymbium to the length of the tibia is approximately 2:1. The cymbium furrow is as long as 1/3 of the cymbium. The tip of the embolus apophysis is slightly pointy. Embolus S-shaped, arising from tegulum at nearly the 6-o’clock-position in ventral view. Conductor arising at 1-o’clock-position from tegulum, elongated, slightly bent. Spermophor slightly S-shaped. RTA arising basally from tibia; base of RTA with a brush of setae. vRTA smaller than dRTA, trapezoidal in retrolateral view. dRTA longer than tibia (Fig. 1A–D).

**Coloration in ethanol**: yellowish. ***Prosoma***: dorsally yellowish, lateral margins dark with yellowish submarginal transverse interval. Labium and gnathocoxae light brownish. Fovea and radial furrow distinctly marked. Sternum yellowish, with margin yellowish brown. Chelicerae deep reddish brown. ***Legs***: yellowish with dark spots. ***Opisthosoma***: dorsally dark khaki covered with dark hairs; ventrally khaki with irregular pattern. Spinnerets yellowish brown (Fig. 1E, F).

**Female** (***paratype***, IZCAS-Ar41605) **Measurements**: PL 8.84, PW 8.39; AW 4.87; OL 9.42, OW 5.51. ***Eyes***: AME 0.3, PME 0.4, ALE 0.55, PLE 0.5, AME-AME 0.37, AME-ALE 0.15, PME-PME 0.57, PME-PLE 0.67, AME-PME 0.52, ALE-PLE 0.6, CHAME 0.17, CHALE 0.45. ***Palp***: 8.5 (2.49, 0.64, 2.11, –, 3.26). ***Legs***: I 27.66 (7.62, 2.62, 7.75, 7.11, 2.56); II 30.42 (8.58, 2.69, 8.9, 7.69, 2.56); III 25.35 (7.24, 2.43, 7.17, 6.02, 2.49); IV 27.86 (7.43, 2.56, 7.82, 7.3, 2.75). Leg formula: II-IV-I-III. **Spination: *Palp***: 131 101 303 2222. ***Legs***: Fe 323, IV 333, Pa 101, Ti I–III 1018, IV 2026, Mt I–III 0004, IV 2026. ***Chelicerae***: Furrow with three anterior teeth, four posterior teeth, and 23 denticles.

***Copulatory organ***: as in diagnosis. Epigynal field wider than long, with two short anterior bands close to the field. Lateral lobes fused. Lobal septum and lateral lobes almost triangular. Glandular appendages are slender and long, the posterior part of internal duct system swollen. Internal ducts half as wide as the epigynal field. Fertilization ducts arising posteriorly. Unexpanded membranous sac between fertilization ducts (Fig. 2A, B).

**Coloration in ethanol**: as in male (Fig. 9A, B).

###### Etymology.

The specific name is dedicated to Mr Hongrui Zhao who collected this species; noun (name) in genitive case.

###### Distribution.

Known only from the type locality (Fig. 10, China, Anhui).

##### Sinopoda jiangzhou

Taxon classificationAnimaliaAraneaeSparassidae

Wang & Li sp. nov.

0D5DD6C8-F009-5E3A-8269-DEA67A1397A3

http://zoobank.org/EAB771F1-EF19-49EC-ACF0-B2573B43D363

**Figure 3.**
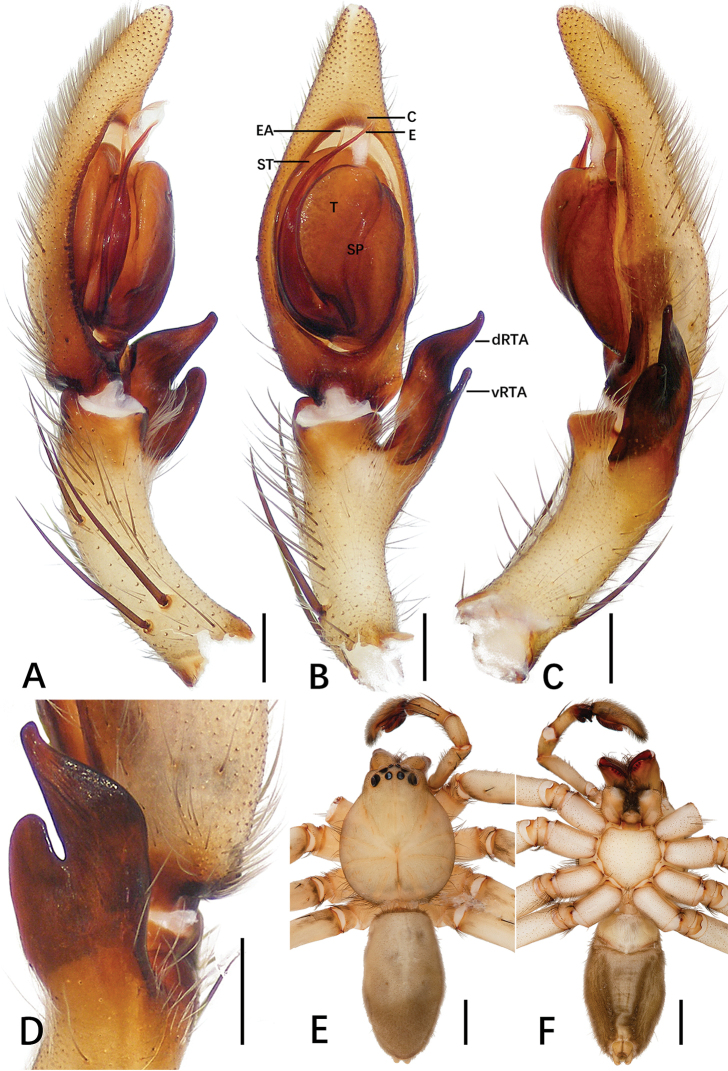
*Sinopoda jiangzhou* sp. nov., holotype male from Underground Gallery **A–C** left palp (**A** prolateral **B** ventral **C** retrolateral) **D** retrolateral view of RTA**E, F** habitus (**E** dorsal **F** ventral). Abbreviations: C conductor, dRTA dorsal branch of retrolateral tibial apophysis, E embolus, EA embolic apophysis, SP spermophor, ST subtegulum, T tegulum, vRTA ventral branch of retrolateral tibial apophysis. Scale bars: 0.5 mm (**A–D**); 2 mm (**E, F**).

**Figure 4.**
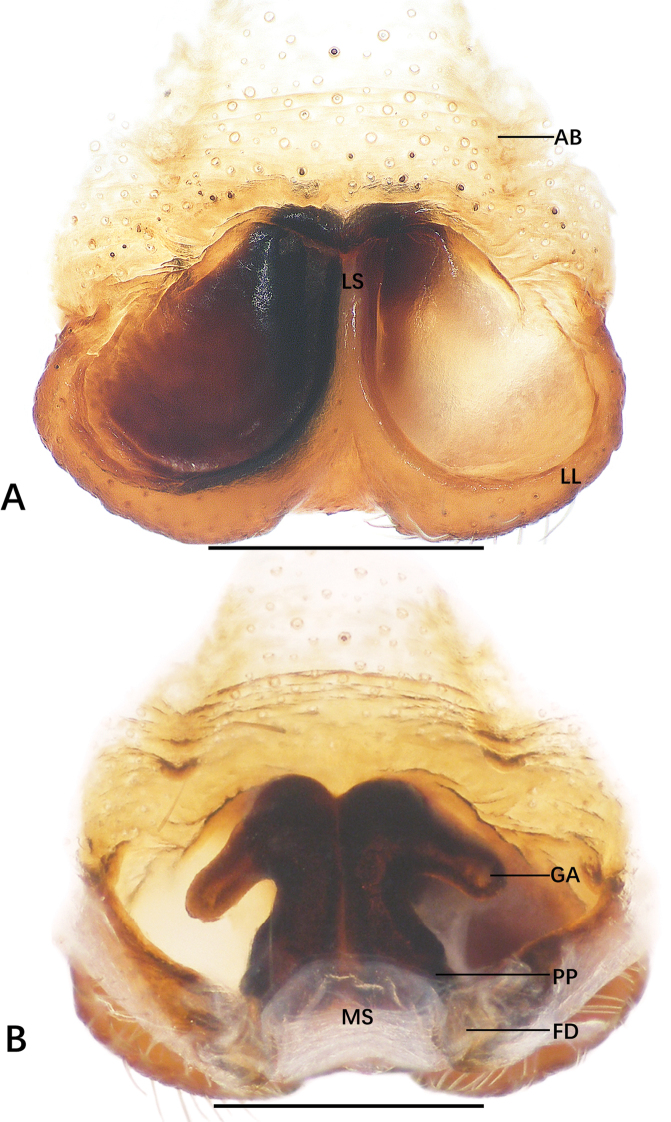
*Sinopoda jiangzhou* sp. nov., paratype female from Underground Gallery **A** epigyne **B** vulva. Abbreviations: AB anterior bands, FD fertilization ducts, GA glandular appendages, LL lateral lobes, LS lobal septum, MS membranous sac, PP posterior part of internal duct system. Scale bars: 0.5 mm.

Figs 3A–F , 4A, B , 9C, D , 10

###### Material examined.

***Holotype*** ♂ (IZCAS-Ar41607), China, Guangxi Zhuang Autonomous Region, Hechi City, Fengshan County, Jiangzhou Village, Underground Gallery; 24.3314°N, 106.9871°E; 449 m; 13 Sept. 2019; Ziyi Wang & Zhigang Chen leg. ***Paratype*** 1 ♂ (IZCAS-Ar41608), same data as holotype. 1 ♀ (IZCAS-Ar41609), same data as holotype, but 25 Mar. 2015; Yunchun Li & Zhigang Chen leg.

###### Diagnosis.

This new species is similar to *Sinopoda tumefacta* Zhong, Jäger, Chen & Liu, 2019 (Zhong et al. 2019: 69, figs 53A–E, 54A–F, 55A–D) in the structure of the embolus and RTA but can be recognized by the following characters: in the male, the conductor is straight and fan-shaped, unlike in *S. tumefacta* (Zhong et al. 2019: fig. 53B) where the conductor is curved and covered by the embolus; the sub-tegulum is noticeably higher in the new species (Fig. 3A–D), but not in *S. tumefacta*; the embolus arises from the tegulum at the 6-o’clock position but at the 5-o’clock position in *S. tumefacta*. The female resembles *S. tumefacta* in the structure of the anterior part of internal ducts and the glandular appendages, which is longer than the posterior part of internal duct system but differs from *S. tumefacta* by: the lateral lobes of the new species (Fig. 4A, B) are narrow, but they are wider in *S. tumefacta* (Zhong et al. 2019: fig. 53D, E); the lobal septum is slender in the new species but broader in *S. tumefacta*.

###### Description.

**Male** (***holotype***, IZCAS-Ar41607) **Measurements**: PL 5.7, PW 4.8; AW 2.43; OL 6.21, OW 3.52. ***Eyes***: AME 0.17, PME 0.23, ALE 0.34, PLE 0.35, AME-AME 0.09, AME-ALE 0.03, PME-PME 0.18, PME-PLE 0.21, AME-PME 0.25, ALE-PLE 0.21, CHAME 0.23, CHALE 0.2. ***Palp***: 9.03 (3.26, 1.41, 1.6, –, 2.76). ***Legs***: I 32.48 (8.78, 2.24, 9.74, 9.03, 2.69); II 35.62 (10.51, 2.05, 10.57, 9.74, 2.75); III 29.15 (8.14, 2.11, 8.33, 7.88, 2.69); IV 30.43 (8.01, 2.11, 8.84, 8.65, 2.82). Leg formula: II-I-IV-III. **Spination: *Palp***: 131, 101, 2101. ***Legs***: Fe 323, IV 123, Pa 101, Ti 2226, Mt I and II 1014, III and IV 2026. ***Chelicerae***: furrow with three anterior teeth, four posterior teeth, and nine denticles.

***Palp***: as in diagnosis. Cymbium longer than tibia. Embolus arising from tegulum at the 6-o’clock position, tip of embolus bent. Embolic apophysis bent at a right angle, slender. Tegulum covering middle of the embolus. Conductor arising from the tegulum at the 1-o’clock-position, elongated straight. Spermophor slightly bent. RTA arising from anterior part of tibia, vRTA smaller than dRTA (Fig. 3B).

**Coloration in ethanol**: yellowish brown. ***Prosoma***: dorsally yellowish brown with fovea and cuticular with a radial pattern. Sternum and ventral coxae pale yellowish brown. Gnathocoxae reddish brown, labium yellowish brown. Chelicerae reddish brown. ***Legs***: light yellowish brown. ***Opisthosoma***: including spinnerets, khaki, sparsely covered with dark hairs (Fig. 3E, F); dorsally with some brown dots and ventrally with two long, distinct furrows posteriorly.

**Female** (***paratype***, IZCAS-Ar41609) **Measurements**: PL 4.23, PW 4.16; AW 2.49; OL 5.96, OW 3.26. ***Eyes***: AME 0.14, PME 0.26, ALE 0.32, PLE 0.34, AME-AME 0.1, AME-ALE 0.06, PME-PME 0.22, PME-PLE 0.36, AME-PME 0.26, ALE-PLE 0.24, CHAME 0.14, CHALE 0.18. ***Palp***: 6.45 (1.66, 1.02, 1.21, –, 2.56). ***Legs***: I 23.12 (6.41, 2.05, 6.47, 6.21, 1.98); II 24.07 (7.05, 2.17, 7.17, 5.44, 2.24); III 22.47 (6.53, 1.85, 6.02, 6.15, 1.92); IV 23.62 (6.66, 1.92, 6.79, 6.08, 2.17). Leg formula: II-I-IV-III. **Spination: *palp***: 131 101 2130 4140. ***Legs***: Fe 323, IV 123, Pa 101, Ti I and II 1018, III 2026, IV 2126, Mt I and II 1014, III and IV 2026. ***Chelicerae***: furrow with three anterior teeth, four posterior teeth, and nine denticles.

***Copulatory organ***: as in diagnosis. Epigynal field wider than long, with short anterior bands. Lateral lobes fused, with wide median incision and distinct, bilobed margin. Fertilization ducts arising posterolaterally. Unexpanded membranous sac between fertilization ducts (Fig. 4A, B).

**Coloration in ethanol**: as in male (Fig. 9C, D).

###### Etymology.

The specific name refers to the type locality, Jiangzhou Village; noun in apposition.

###### Distribution.

Known only from the type locality (Fig. 10, China, Guangxi).

##### Sinopoda saiyok

Taxon classificationAnimaliaAraneaeSparassidae

Wang & Li sp. nov.

9B5964B2-2B55-5755-A111-642BE402B784

http://zoobank.org/329FFEC6-0A28-44AB-A33F-1383DD9D1CC1

**Figure 5.**
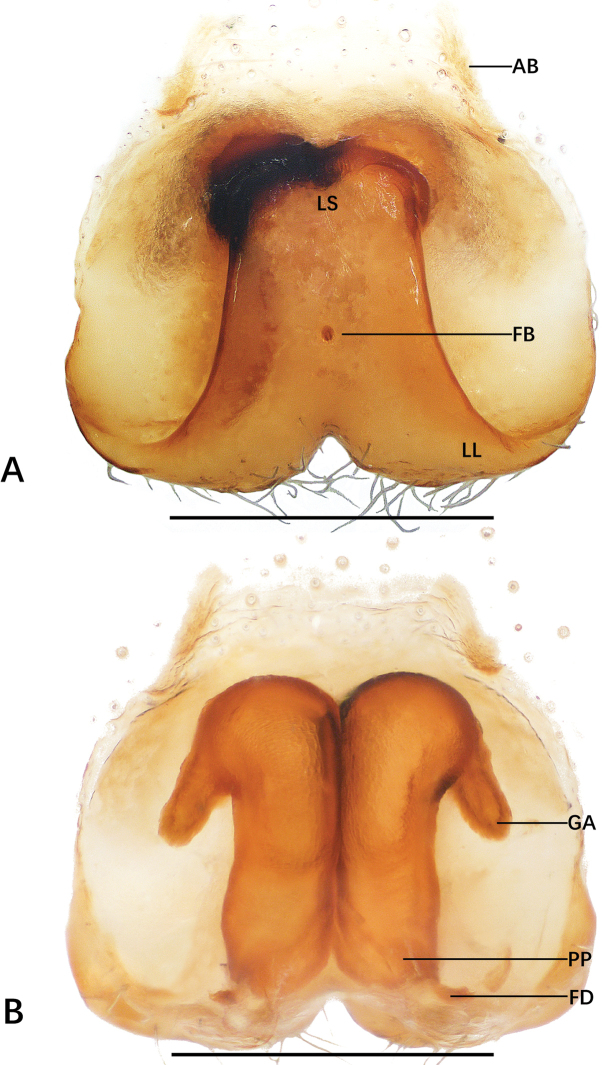
*Sinopoda saiyok* sp. nov., holotype female from Sai Yok District **A** epigyne **B** vulva. Abbreviations: AB anterior bands, FB fusion bubble, FD fertilization ducts, GA glandular appendages, LL lateral lobes, LS lobal septum, PP posterior part of internal duct system. Scale bars: 0.5 mm.

Figures 5A, B , 9E, F , 10

###### Material examined.

***Holotype*** ♀ (IZCAS-Ar41647), Thailand, Kanchanaburi Province, Sai Yok District, Wang Krachae Subdistrict, unnamed cave; 14.2036°N, 99.0277°E; 82 m; 11 January 2014; Prasit Wongprom leg.

###### Diagnosis.

This new species resembles *Sinopoda bifurca* Grall & Jäger, 2020 (Grall and Jäger 2020: 11, fig. 4d, e) in having similar lateral lobes, but it can be recognized by the uniquely rectangular lobal septum and the reduced posterior part of internal duct system (Fig. 5A, B), whereas the posterior part of internal duct system slightly swollen in *S. bifurca*.

###### Description.

**Female** (***holotype***, IZCAS-Ar41647) **Measurements**: PL 3.28, PW 3.24; AW 1.88; OL 4.24, OW 2.64. ***Eyes***: AME 0.12, PME 0.08, ALE 0.14, PLE 0.16, AME-AME 0.10, AME-ALE 0.05, PME-PME 0.18, PME-PLE 0.22, AME-PME 0.13, ALE-PLE 0.15, CHAME 0.11, CHALE 0.15. ***Palp***: 4.72 (1.53, 0.44, 1.34, –, 1.41). ***Legs***: I 15.04 (4.10, 1.66, 4.16, 3.84, 1.28); II 17.61 (5.06, 1.98, 4.93, 4.23, 1.41); III 15.18 (4.23, 1.73, 4.23, 3.65, 1.34); IV 15.43 (4.42, 1.41, 4.10, 3.97, 1.53). Leg formula: II-IV-III-I. **Spination: *palp***: 131 101 2130 3030. ***Legs***: Fe I–IV 323, Pa I–IV 111, Ti I–IV 2026, Mt I–IV 2026. ***Chelicerae***: furrow with three anterior teeth, four posterior teeth, and without denticles.

***Copulatory organ***: as in diagnosis. Epigynal field slightly wider than long, with two short anterior bands slightly fused with field, with one fusion bubble medially. The width of the lobal septum is equal to 1/3 the width of the epigynal field. The lobal septum is partly fused to the epigynal field. The anterior part of the internal ducts is discernibly swollen. The glandular appendages are blunt and bent at a right angle, extending laterally in posterior half of internal duct system. Internal duct system fused along whole median line. The posterior part of the internal duct system are miniaturized and narrower than anterior part of internal ducts and with the fertilization ducts arising posterolaterally. Unexpanded, membranous sac between fertilization ducts (Fig. 5A, B).

**Coloration in ethanol**: yellowish brown. ***Prosoma***: dorsally yellowish brown with fovea and cuticular with a radial, yellowish-brown pattern. Sternum and ventral coxae pale yellowish brown, gnathocoxae deep yellowish brown, labium reddish brown. Chelicerae deep reddish brown. ***Legs***: yellowish brown. ***Opisthosoma***: including spinnerets, greyish brown to yellowish brown, sparsely covered with brown hairs (Fig. 9E, F).

**Male**: unknown.

###### Etymology.

The specific name refers to the type locality, Sai Yok District; noun in apposition.

###### Distribution.

Known only from the type locality (Fig. 10, Thailand, Kanchanaburi).

##### Sinopoda yanjin

Taxon classificationAnimaliaAraneaeSparassidae

Wang & Li sp. nov.

3FD2C966-917B-5221-BB44-2BF82B5A0023

http://zoobank.org/85904853-5C78-4507-A8F7-566A075721C8

**Figure 6.**
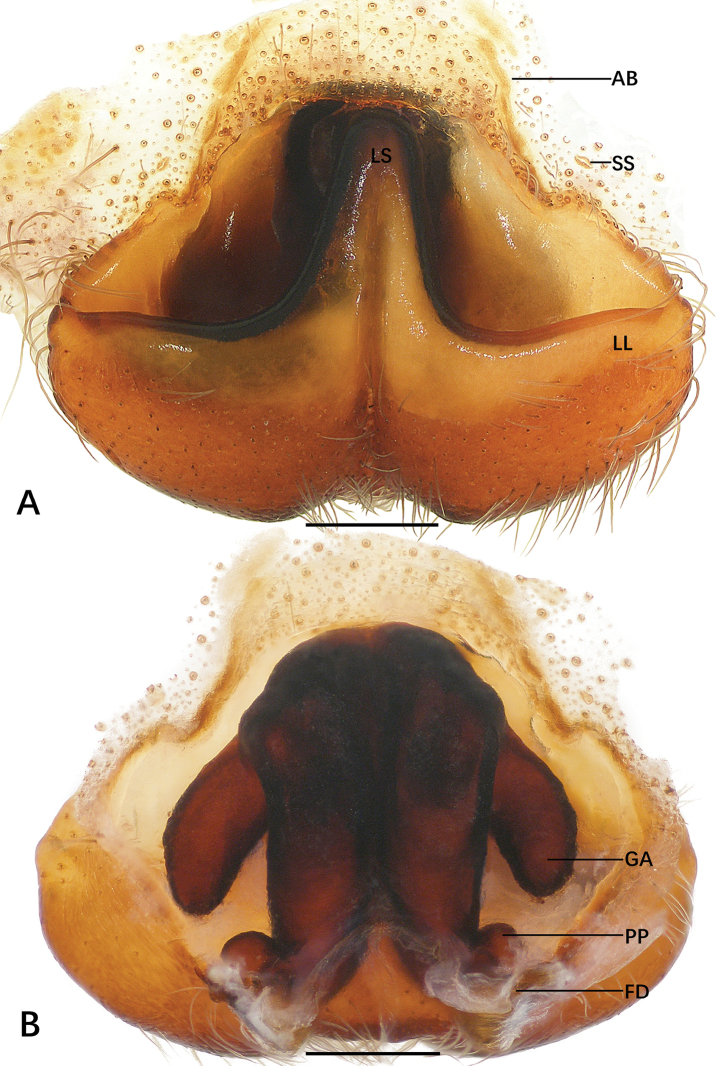
*Sinopoda yanjin* sp. nov., holotype female from Yanjin County **A** epigyne **B** vulva. Abbreviations: AB anterior bands, FD fertilization ducts, GA glandular appendages, LL lateral lobes, LS lobal septum, PP posterior part of internal duct system, SS slit sensillum. Scale bars: 0.5 mm.

Figs 6A, B , 9G, H , 10

###### Material examined.

***Holotype*** ♀ (IZCAS-Ar41610), China, Yunnan Province, Zhaotong City, Yanjin County, Doushaguan Town, near Xiangshui Cave, unnamed cave; 28.0381°N, 104.07986°E; 774 m; 15 March 2015; Yunchun Li & Jinchen Liu leg. ***Paratypes*** 4 ♀ (IZCAS-Ar41611 to IZCAS-Ar41614); China, Yunnan Province, Zhaotong City, Yanjin County, Doushaguan Town, Wuchidao Scenic Area; 28.0398°N, 104.1150°E; 548 m; 19 Sept. 2020; Ziyi Wang leg.

###### Diagnosis.

This new species can be separated from other *Sinopoda* species by the unique arrow-shaped lobal septum; the internal duct system is conspicuously swollen and broad; the width of the glandular appendages is equal to the width of medial part of the internal ducts (Fig. 6A, B).

###### Description.

**Female** (***holotype***, IZCAS-Ar41610) **Measurements**: PL 8.71, PW 8.52; AW 4.87; OL 10.83, OW 7.24. ***Eyes***: AME 0.34, PME 0.4, ALE 0.5, PLE 0.48, AME-AME 0.3, AME-ALE 0.36, PME-PME 0.48, PME-PLE 0.72, AME-PME 0.58, ALE-PLE 0.64, CHAME 0.2, CHALE 0.4. ***Palp***: 12.16 (3.46, 1.85, 2.62, –, 4.23). ***Legs***: I 34.72 (9.67, 3.97, 9.42, 8.46, 3.20); II 37.21 (11.08, 4.23, 10.06, 8.90, 2.94); III 31.59 (9.23, 3.78, 8.78, 6.98, 2.82); IV 34.84 (9.99, 3.71, 8.52, 9.16, 3.46). Leg formula: II-IV-I-III. **Spination: *palp***: 131 101 2120 2030. ***Legs***: Fe I–III 323, IV 333, Pa I–IV 101, Ti I–IV 2224, Mt I–III 2024, IV 3034. ***Chelicerae***: Furrow with three anterior teeth, four posterior teeth, and 16 denticles.

***Copulatory organ***: as in diagnosis. Epigynal field wider than long, with one long anterior band partly integrated into the field and one slit sensillum on each side, close to the field. The lobal septum is not fused with epigynal field and has a distinct indentation medially. Lateral lobes fused, with median indentation. The anterior part of the internal ducts is wider than the posterior part. The glandular appendages are blunt and wide, extending posteriorly to the posterior half of the internal duct system. The width of the glandular appendages is equal to the width of medial part of the internal ducts. Lateral furrow partly fused, inconspicuous. The posterior part of internal duct system bulging slightly laterally, fertilization ducts arising posteriorly from the posterior part of the internal duct system. Unexpanded membranous sac between fertilization ducts (Fig. 6A, B).

**Coloration in ethanol**: brown. ***Prosoma***: dorsally reddish brown with distinct radial furrow and fovea, sparsely covered with dark hairs. Labium and gnathocoxae deep reddish brown, with dark margin. Sternum bright yellowish brown, with reddish brown margin. ***Legs***: khaki, with distal parts darker, covered with dark hairs. Chelicerae dark reddish brown. ***Opisthosoma***: dorsally and ventrally reddish, slightly brownish, with an irregular pattern; ventrally with two longitudinal red lines between epigastric furrow and spinnerets. Spinnerets khaki (Fig. 9G, H).

**Male**: unknown.

###### Etymology.

The specific name is taken from the type locality, Yanjin County; noun in apposition.

###### Distribution.

Known only from the type locality (Fig. 10, China, Yunnan).

##### Sinopoda yanzi

Taxon classificationAnimaliaAraneaeSparassidae

Wang & Li sp. nov.

E5F76E98-79BB-510D-9242-DCA792DFBAB4

http://zoobank.org/4D942E8A-5EC2-4FE9-BB10-005EF4F8ADAB

**Figure 7.**
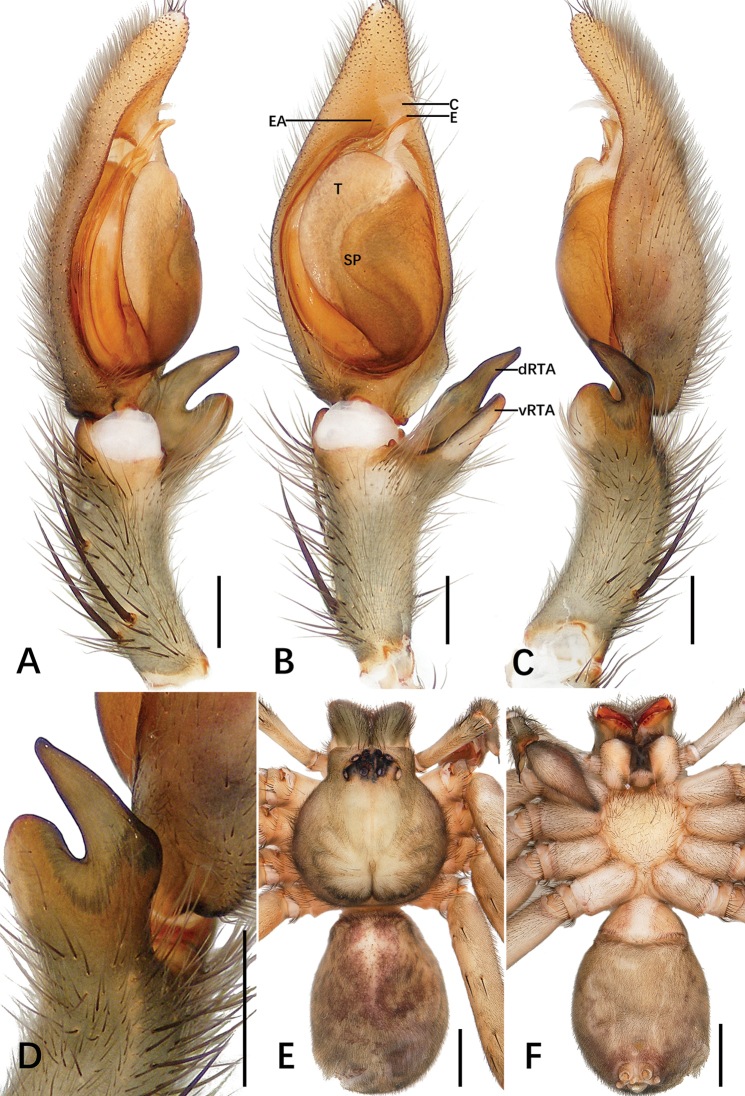
*Sinopoda yanzi* sp. nov., holotype male from Yanzi Cave **A–C** left palp (**A** prolateral **B** ventral **C** retrolateral) **D** retrolateral view of RTA**E, F** habitus (**E** dorsal **F** ventral). Abbreviations: C conductor, dRTA dorsal branch of retrolateral tibial apophysis, E embolus, EA embolic apophysis, SP spermophor, T tegulum, vRTA ventral branch of retrolateral tibial apophysis. Scale bars: 0.5 mm (**A–D**); 2 mm (**E, F**).

**Figure 8.**
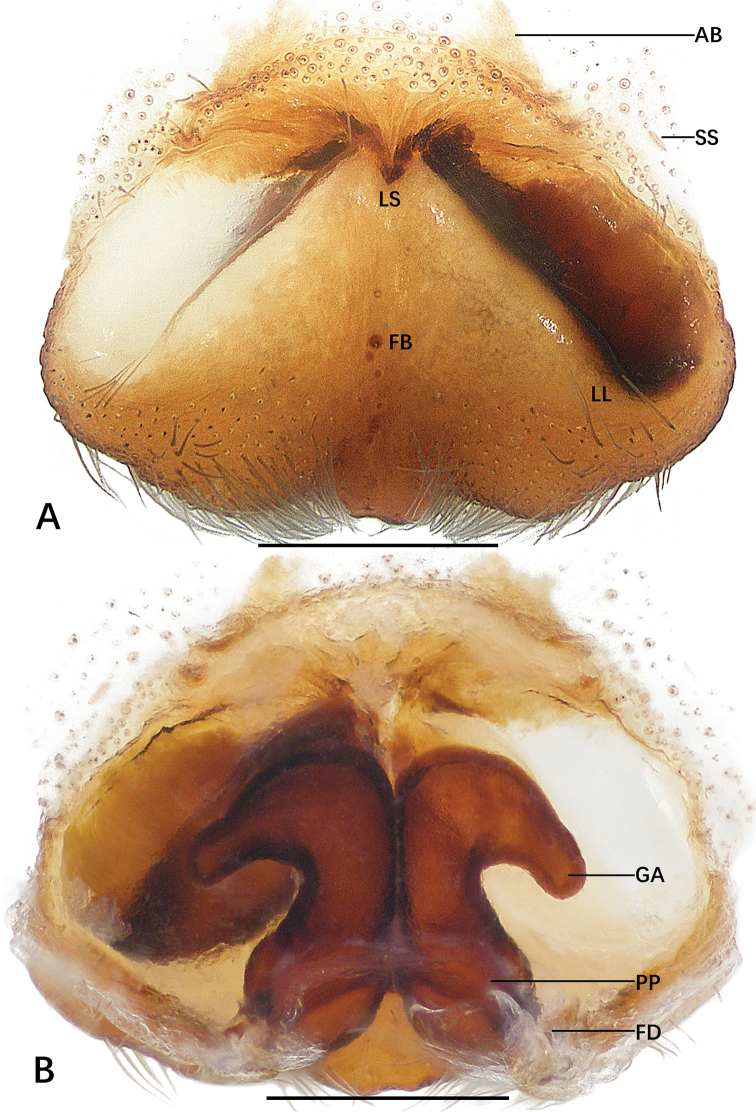
*Sinopoda yanzi* sp. nov., paratype female from Yanzi Cave **A** epigyne **B** vulva. Abbreviations: AB anterior bands, FB fusion bubble, FD fertilization duct, GA glandular appendages, LL lateral lobes, LS lobal septum, MS membranous sac, PP posterior part of internal duct system, SS slit sensillum. Scale bars: 0.5 mm.

**Figure 9.**
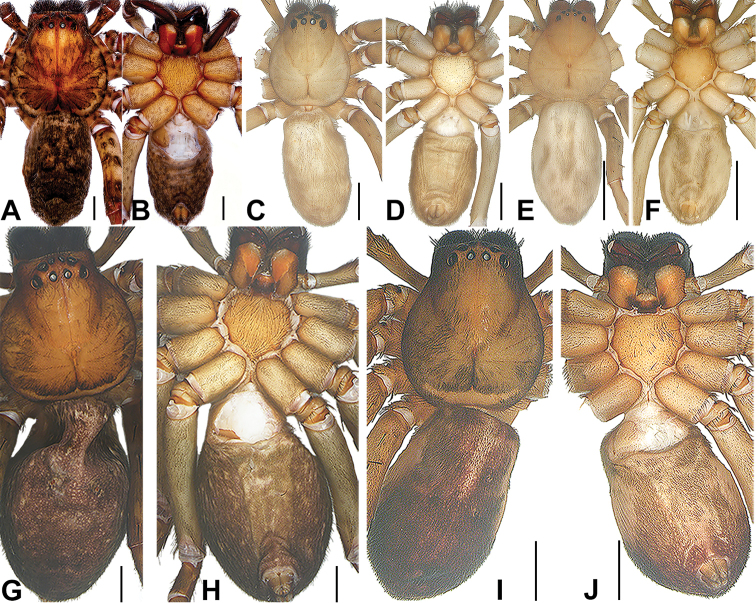
**A, B***S. hongruii* sp. nov. female paratype **C, D***S. jiangzhou* sp. nov. female paratype **E, F***S. saiyok* sp. nov. female holotype **G, H***S. yanjin* sp. nov. female holotype **I, J***S. yanzi* sp. nov. female paratype. Scale bars: 2 mm.

**Figure 10.**
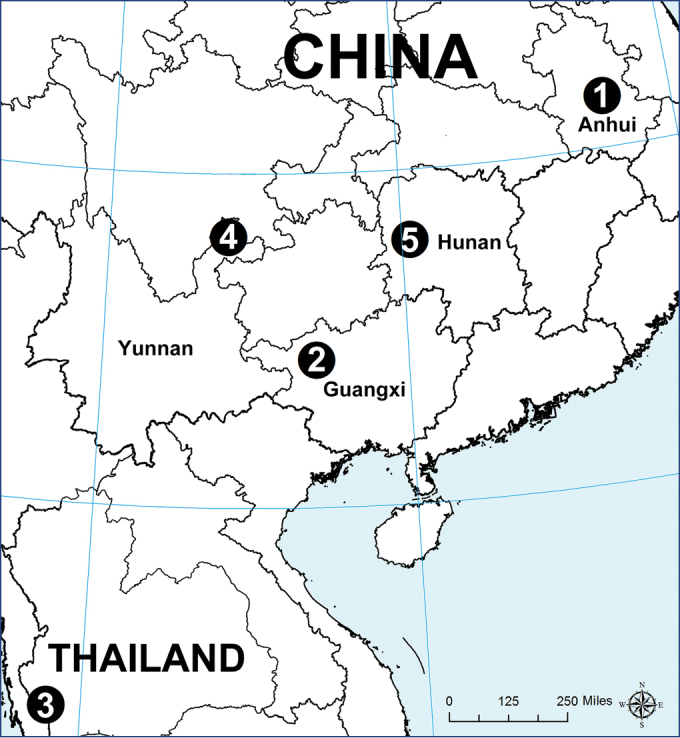
Locality records for five new species of *Sinopoda*: **1***S. hongruii* sp. nov. (Anhui, China) **2***S. jiangzhou* sp. nov. (Guangxi, China) **3***S. saiyok* sp. nov. (Kanchanaburi, Thailand) **4***S. yanjin* sp. nov. (Yunnan, China) **5***S. yanzi* sp. nov. (Hunan, China).

Figures 7A–F , 8A, B , 9I, J , 10

###### Material examined.

***Holotype*** ♂ (IZCAS-Ar41615), China, Hunan Province, Huaihua City, Chenxi County, Huomachong Town, Yanzi Cave; 27.8545°N, 110.2605°E; 408 m; 6 Sept. 2019, Ziyi Wang & Zhigang Chen leg. ***Paratype*** 1 ♀ (IZCAS-Ar41627), same data as holotype, but 18 Mar. 2016; Yulong Li & Zhigang Chen leg. 1 ♀ (IZCAS-Ar41628), same data as holotype, 6 Sept. 2019; Ziyi Wang & Zhigang Chen leg.

###### Diagnosis.

The male of this new species is similar to *Sinopoda tumefacta* Zhong, Jäger, Chen & Liu (Zhong et al. 2019: 69, figs 53A–E, 54A–F, 55A–D) in the shape of conductor, but it can be distinguished by the following: the dRTA is sharp, short, and triangular, while the dRTA is long and an irregular-quadrilateral in *S. tumefacta*; the vRTA is smooth in ventral view (Fig. 7A–D), while the vRTA is concave in ventral view in *S. tumefacta*. The female of this new species is similar to *S. dehiscens* Zhong, Jäger, Chen & Liu, 2019 (Zhong et al. 2019: 28, figs 20A, B, 21A–D) in having an analogous lobal septum and lateral lobes, but it can be separated by the following: the middle part of lateral lobes has a downward protrusion but there is no protrusion in *S. dehiscens*; the anterior part of the internal ducts is not fused with the median line, while in *S. dehiscens* the ducts are distinctly divided; the glandular appendages are wider than the posterior parts of the internal duct system in this new species, but the glandular appendages are as wide as the posterior parts of internal duct system in *S. dehiscens*; the posterior parts of internal duct system are swollen and slightly divided, while they are distinctly separated posterolaterally in *S. dehiscens*; this new species has fusion bubbles medially on the lobal septum, but *S. dehiscens* has no fusion bubble (Fig. 8A, B).

###### Description.

**Male** (***holotype***, IZCAS-Ar41615) **Measurements**: PL 5.44, PW 4.87; AW 2.88; OL 6.08, OW 3.91. ***Eyes***: AME 0.18, PME 0.26, ALE 0.3, PLE 0.32, AME-AME 0.24, AME-ALE 0.04, PME-PME 0.3, PME-PLE 0.34, AME-PME 0.4, ALE-PLE 0.28, CHAME 0.2, CHALE 0.28. ***Palp***: 8.12 (2.69, 1.02, 1.66, –, 2.75). ***Legs***: I 25.11 (7.05, 1.92, 7.37, 6.66, 2.11); II 28.44 (8.14, 2.05, 8.07, 7.69, 2.49); III 22.42 (6.73, 1.98, 6.34, 5.32, 2.05); IV 24.01 (6.6, 1.79, 6.6, 6.85, 2.17). Leg formula: II-I-IV-III. **Spination: *palp***: 131 101 – 3010. ***Legs***: Fe 323, IV 123, Pa 101, Ti I and II 1318, III and IV 1216, Mt 1014, III 2024, IV 2026. ***Chelicerae***: furrow with three anterior teeth, four posterior teeth, and six denticles.

***Palp***: as in diagnosis. Cymbium almost twice as long as tibia. Embolus arising from tegulum in nearly the 5-o’clock-position. Embolic tip slightly longer than the embolic apophysis. Conductor arising from tegulum at the 1-o’clock-position, elongated flake with distal part flat. Tegulum covers medial part of embolus. Spermophor distinctly S-shaped. RTA arising from anterior part of tibia (Fig. 7B).

**Coloration in ethanol**: yellowish brown. ***Prosoma***: dorsally yellowish brown with distinct fovea and radial furrow, covered with dark hairs. Labium and sternum yellowish brown. Chelicerae deep reddish brown. ***Legs***: yellowish brown. ***Opisthosoma***: dorsally dark reddish brown, covered with dark hairs, with bright bands in anterior part; ventrally yellowish brown with bright band on both sides of central axis. Spinnerets yellowish brown (Fig. 7E, F).

**Female** (***paratype***, IZCAS-Ar41627) **Measurements**: PL 5.83, PW 5.32; AW 3.46; OL 7.05, OW 4.55. ***Eyes***: AME 0.2, PME 0.22, ALE 0.3, PLE 0.32, AME-AME 0.22, AME-ALE 0.3, PME-PME 0.4, PME-PLE 0.48, AME-PME 0.32, ALE-PLE 0.1, CHAME 0.12, CHALE 0.24. ***Palp***: 8.62 (2.49, 1.02, 1.85, –, 3.26). ***Legs***: I 21.25 (6.08, 2.56, 5.76, 4.93, 1.92); II 22.73 (6.73, 2.75, 6.21, 5.12, 1.92); III 20.16 (6.15, 2.43, 5.12, 4.8, 1.66); IV 21.65 (6.6, 2.24, 5.51, 5.25, 2.05). Leg formula: II-IV-I-III. **Spination: *palp***: 131 101 213 3030. ***Legs***: Fe I and II 323, III 333, IV 133, Pa 101, IV 000, Ti I and II 1018, III 2026, IV 2126, Mt 1014, IV 3034. ***Chelicerae***: furrow with three anterior teeth, four posterior teeth, and 28 denticles.

***Copulatory organ***: as in diagnosis. Epigynal field wider than long, with one short anterior band partly integrated with the field and one slit sensillum on each side close to the field. Lateral lobes fused, concave medially. Anterior and posterior part of internal ducts not fused along median line. Glandular appendages extending laterally in anterior half of internal duct system. Posterior part of internal duct system swollen, fertilization ducts arising posteriorly. Unexpanded membranous sac between fertilization ducts (Fig. 8A, B).

**Coloration in ethanol**: as in male, but dorsal prosoma yellowish brown, and posterior part with a bright band (Fig. 9I, J).

###### Etymology.

The specific name refers to the type locality, Yanzi Cave; noun in apposition.

###### Distribution.

Known only from the type locality (Fig. 10, China, Hunan).

## Supplementary Material

XML Treatment for Sinopoda hongruii

XML Treatment for Sinopoda jiangzhou

XML Treatment for Sinopoda saiyok

XML Treatment for Sinopoda yanjin

XML Treatment for Sinopoda yanzi
